# Effects of perfusion processes under limiting conditions on different Chinese Hamster Ovary cells

**DOI:** 10.1186/1753-6561-7-S6-P64

**Published:** 2013-12-04

**Authors:** Anica Lohmeier, Tobias Thüte, Stefan Northoff, Jeff Hou, Trent Munro, Thomas Noll

**Affiliations:** 1 Institute of Cell Culture Technology, Bielefeld University, Germany; 2 TeutoCell AG, Bielefeld, Germany; 3 The Australian Institute for Bioengineering and Nanotechnology (AIBN), University of Queensland, Brisbane, Australia; 4 Center for Biotechnology (CeBiTec), Bielefeld University, Germany

## Background

The use of perfusion culture to generate biopharmaceuticals is an attractive alternative to fed-batch bioreactor operation. The process allows for generation of high cell densities, stable culture conditions and a short residence time of active ingredients to facilitate the production of sensitive therapeutic proteins.

However, challenges remain for efficient perfusion based production at industrial scale, primarily complexity of required equipment and strategies adopted for downstream processing. For perfusion systems to be industrially viable there is a need to increase product yields from a perfusion-based platform.

We have shown previously that one effective way to enhance the cell specific productivity is via glucose limitation [1,2]. The mechanisms leading to an increased productivity under these glucose limiting conditions are still under investigation. Preliminary studies using proteomic analysis have indicated changes in histone acetylation [2].

In this work, we investigated the influence of glucose limited conditions on the production of two different recombinant proteins in perfusion processes.

## Materials and methods

CHO-MUC2 and CHO-XL99 cell lines were cultivated perfusion based in a 2 L pO_2_- and pH-controlled bioreactor using an internal spin filter (20 μm) for cell retention. In addition these cell lines were cultivated both under limiting and non-limiting glucose conditions in fed-batch mode in a four vessel parallel single-use system (Bayshake, Bayer Technology Services GmbH).

Perfusion mode was started three days after inoculation; flow rate was adjusted between 0.3 d^-1 ^and 0.6 d^-1^. For fed-batch cultivation the limiting range for glucose concentration was chosen between 0.2 and 0.5 g/L. Reference cultivation was performed between 1.5 and 3.0 g/L. Both cultures were fed with similar volumes.

All cultivations were performed in chemically-defined, animal-component free CHO growth media (TeutoCell AG).

Viable cell density and viability were determined using the automated cell counting system CEDEX (Roche Diagnostics), glucose and lactate concentrations were detected via YSI (YSI life sciences). Amounts of IgG1 were quantified via Protein A HPLC, anti IL-8 mAb purified from a CHO DP-12 cell clone was used as a standard. Mucin-2 quantity was measured via photometric quantification of eGFP coupled to the Mucin 2.

## Results

**Figure 1:**
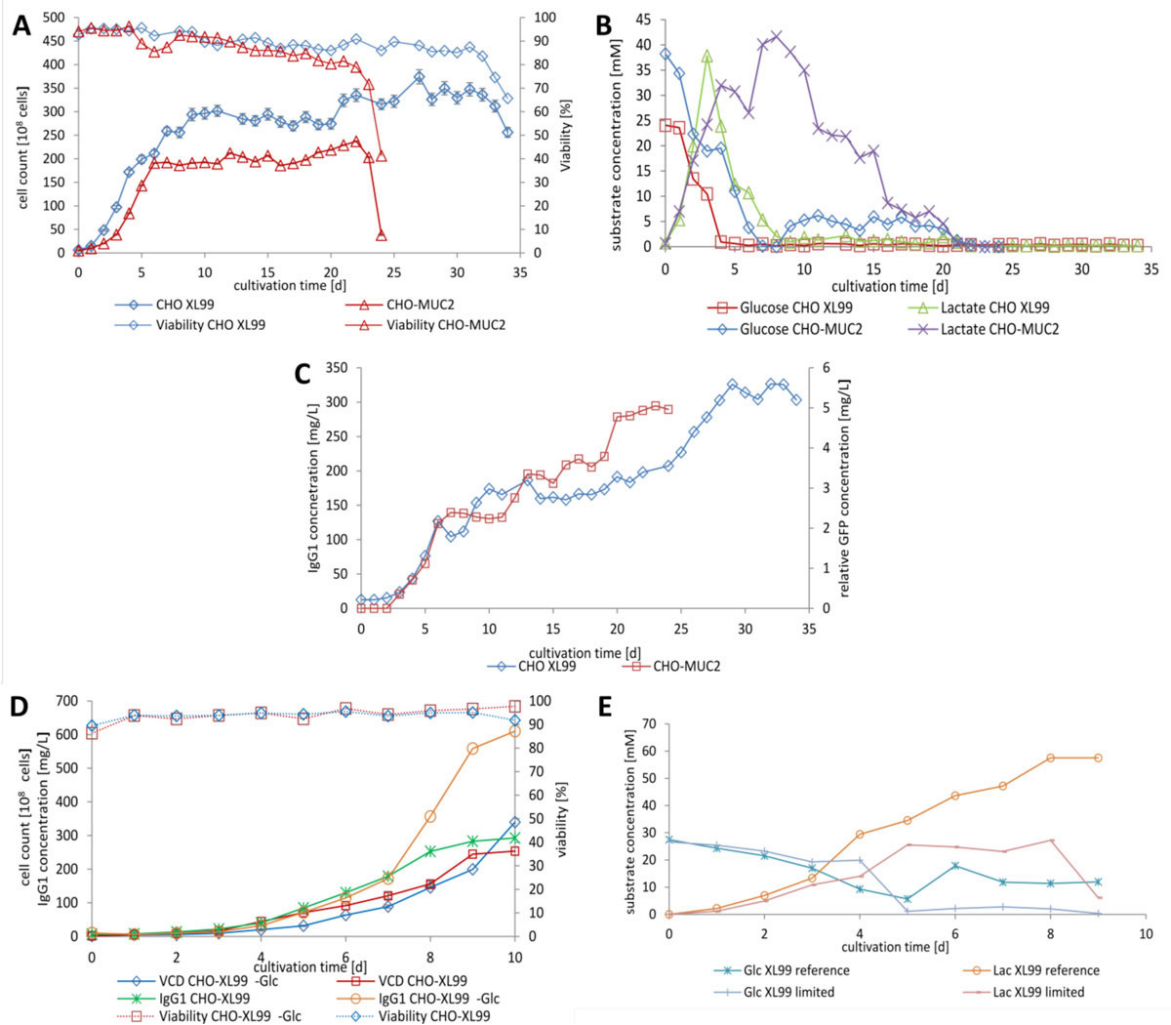
**A** Viable cell counts and cell viabilities for the time course of CHO-MUC2 and CHOXL99 cells during perfusion process; **B**: glucose and lactate concentrations during CHO-XL99 and CHO-MUC2 perfusion cultivation; **C**: Concentration of IgG1 mAb and eGFP during CHO perfusion cultivations; **D**: Viable cell counts and mAb concentration for the time course of CHO-XL99 fed-batch cultivations; **E**: Glucose and lactate concentrations for the time course of CHO-XL99 under limiting (-Glc) and non-limiting conditions.

Using perfusion mode with a 20 μm spin filter as cell retention device we have reached viable cell densities of 1.4·10^7 ^cells/mL in a 24 day perfusion run of CHO-MUC2 (Figure 1A). During perfusion the average viability remained higher than 85% was attained. After 6 days of cultivation glucose reached a limiting concentration below 1 mM (Figure 1B). Meanwhile a relative eGFP concentration of 5 mg/L was achieved (Figure 1C) and cell specific productivity increased by 90% during glucose limitation (data not shown).

A further 34 day perfusion cultivation using a CHO-XL99 clone reached a viable cell density of 2.6·10^7 ^cells/mL with an average viability of 90% (Figure 1A). Glucose and Lactate concentrations of CHO-XL99 were below detectable limits on day 8 and 17 post-inoculation respectively (Figure 1B). Simultaneously, cells were able to reach an IgG1 titer of 326 mg/L, with significant increases in product titer observed after 24 days of culture (Figure 1C). Simultaneously, cell specific productivity showed a slight increase after 25 days (data not shown).

Neither the CHO-MUC2, nor the CHO-XL99 cells showed any limitations concerning other substrates, e.g. amino acids (data not shown).

In two parallel fed-batch cultivations of the CHO-XL99 clone the glucose limited culture showed similar growth characteristics as the unlimited reference culture. Viable cell densities of 1.9·10^7 ^cells/mL (reference) and 2.9·10^7 ^cells/mL (-Glc), respectively, were observed (Figure 1D). The limited culture reached an IgG1 concentration of 610 mg/L, in contrast to 292 mg/L produced by the reference culture (Figure 1D). Under glucose limitation the cells consumed lactate while under non-limiting conditions lactate accumulated (Figure 1E).

## Conclusions

During perfusion processes under glucose limitation three characteristic phases appear: At first glucose concentration is high and lactate is below detection limit. Afterwards glucose is metabolized into lactate with an increasing lactate formation rate. In the end both metabolites are consumed and an increase in product concentration and cell specific productivity occurs.

Reduced lactate formation was observed during the perfusion run as CHO-MUC2 cells shift towards a more efficient glucose metabolism. Thereby cell specific productivity of CHO-MUC2 cells increased by 90% during glucose limitation.

CHO-XL99 cells showed a similar metabolic shift during perfusion along with increased mAb production as well as in fed-batch cultivation. Resulting from this fed-batch cultivations allow predictions concerning cell behavior under glucose limitation in perfusion.

To analyse the impact of limiting conditions on transcriptome level of CHO cells, a microarray will be used. This proprietary CHO microarray contains 41.304 different probes to elucidate reasons for the increase in cell specific productivity.
